# Cell population data in identifying active tuberculosis and community-acquired pneumonia

**DOI:** 10.1515/med-2021-0322

**Published:** 2021-08-11

**Authors:** Tingting Sun, Bin Wu, Zhonglan Luo, Jing Wang, Shaoli Deng, Qing Huang

**Affiliations:** Department of Laboratory Medicine, Daping Hospital, Army Medical University, Chongqing, 400042, People’s Republic of China; Department of Laboratory Medicine, Chongqing Public Health Medical Center, Chongqing, 400036, China

**Keywords:** active tuberculosis, cell population data, community-acquired pneumonia, VCS technology, CPD parameters

## Abstract

**Objective:**

Leukocyte morphological parameters known as CPD (cell population data) is detected by hematology analyzer UniCel DxH800 with VCS technology. This study aimed to investigate the diagnostic efficacy of morphological changes in CPD parameters in distinguishing active tuberculosis from community-acquired pneumonia.

**Methods:**

From October 2018 to February 2019, 88 patients with active tuberculosis, 78 patients with community-acquired pneumonia, and 89 healthy controls were enrolled in this study. CPD was obtained using Unicel DxH800 analyzer for all whole blood samples, one-way ANOVA (non-parametric) and area analysis under ROC curve were performed.

**Results:**

The neutrophil mean conductivity (NMC), monocyte mean volume (MMV), monocyte mean conductivity (MMC), lymphocyte percentage (LY%), and monocyte percentage (MO%) were significantly higher in the active tuberculosis group than in the community-acquired pneumonia group. The white blood cell (WBC) count and neutrophil percentage (NE%) were significantly lower in the active tuberculosis group than in the community-acquired pneumonia group. The analysis of the area under the ROC curve proved that WBC count, neutrophil percentage (NE%), lymphocyte percentage (LY%), and monocyte percentage (MO%) did not achieve a higher area under the curve (AUC: 0.63, 0.71, 0.62, and 0.7, respectively). However, the AUC of NMC, MMV, and MMC in the CPD parameters was 0.951, 0.877, 0.98, respectively, and the simultaneous measurement of the three parameters was 0.99. The sensitivity and specificity were 98.5% and 91.1%, respectively.

**Conclusion:**

The combined diagnosis of NMC, MMV, and MMC could assist the clinical diagnosis of active tuberculosis and community-acquired pneumonia.

## 1 Introduction

Active tuberculosis (ATB) and community-acquired pneumonia (CAP) have similar symptoms, such as fever, cough, and expectoration; also, similarities are found in the imaging findings of pulmonary inflammation [1]. When the two diseases are combined, the patient’s condition will become more complicated, increasing the difficulty of identification and diagnosis. The two diseases often cover each other’s symptoms. The symptoms of the first disease are more obvious, which may cause missed diagnosis and misdiagnosis of another disease. There are many cases of tuberculosis as the first disease with secondary pneumonia, while the cases of pneumonia as the first, causing neglected treatment of tuberculosis reports are seldom. Currently, distinguishing the two diseases clinically is still difficult [2]. Therefore, a simple, fast, and accurate method is needed to distinguish ATB from CAP. The cell population data (CPD) can measure the inherent biological characteristics of more than 8,000 white blood cells simultaneously using the VCS technology of the blood analyzer UniCel DxH800. The VCS technology uses the direct current impedance to detect cell volume (V), the exact size of all cell types, and the conductivity of the internal structure of the cell (C) by radiofrequency opacity. Multiple angles light scatter (S) includes upper medium angle light scatter (UMALS), medium angle light scatter (MALS), lower medium angle light scatter (LMALS), and lower angle light scatter (LALS) to detect cell particle size and membrane morphology and axial light loss (AL2) so as to analyze cell transparency. Leukocytes are detected and analyzed by simultaneously obtaining three morphological parameters directly related to cell morphology. In three-dimensional space, a series of parameters can be obtained by comprehensively measuring the cell volume, size, and particle size, which can be accurately and quickly used to perform a microscopic evaluation of the WBC morphology [3,4,5]. In this study, the morphological changes in leukocyte CPD parameters were evaluated to investigate its diagnostic efficacy in distinguishing active tuberculosis from community-acquired pneumonia.

## 2 Material and methods

### 2.1 Case selection

This study included 88 patients with ATB from Chongqing Infectious Disease Medical Center from October 2018 to February 2019, 78 patients including CAP inpatients in the Department of Laboratory Medicine, Daping Hospital, Army Medical University, and 89 healthy controls who visited the physical examination center for health checkups.

The inclusion criteria for patients with ATB, in accordance with the “Public Health Organization Standards of the People’s Republic of China for the diagnosis of tuberculosis WS 288-2017,” were as follows: sputum culture, sputum antacid staining, at least one positive molecular diagnosis, and chest X-ray findings consistent with TB imaging lesions. Selected patients were all newly diagnosed patients with no history of anti-TB treatment.

The admission criteria for CAP and the diagnosis complied with the guidelines for the diagnosis and treatment of acquired pneumonia issued by the Respiratory Branch of the Chinese Medical Association in 2016 [6]. The criteria were as follows: (1) community incidence; (2) pneumonia-related clinical manifestations: (i) new symptoms of cough, sputum, or pre-existing respiratory disease worsened, with or without chest pain, and purulent sputum, (ii) signs of consolidation of the lungs and/or smell of wet rales, (iii) fever, (iv) the number of peripheral blood leukocytes >10 × 10^9^/L or <4 × 10^9^/L, with or without a nuclear shift to the left; and (3) chest imaging showing newly appearing patchy infiltrates, lobes, or segments with consolidated shadows, ground-glass opacity, or interstitial changes with or without pleural effusion. Patients with any of the aforementioned criteria who were diagnosed with bacterial pneumonia through bacterial staining and culture, excluding lung tumors, TB, noninfectious pulmonary interstitial disease, pulmonary embolism, pulmonary edema, atelectasis, pulmonary eosinophil infiltration, and pulmonary vasculitis.

The inclusion criteria for the healthy control group were as follows: individuals who went to the hospital for physical examination during the same period; no history of exposure to patients with TB; normal chest X-ray; negative results for interferon-gamma release assay T-SPOT; and no fever and cough. The liver function, kidney function, hepatitis B, and routine blood tests were normal.

The exclusion criteria were as follows: age <18 years; pregnant women; HIV infection; cancer; patients with severe abnormalities in liver and kidney function; patients with autoimmune diseases; patients who received immunosuppressants such as glucocorticoids; or patients who received anti-TB treatment.

**Ethical approval:** The study protocol was approved by the hospital ethics committee.

### 2.2 Testing method

#### 2.2.1 CPD parametric analysis

All samples were collected in EDTA-anticoagulated tubes by the experienced phlebotomists and analyzed within 4 h after specimen collection using UniCel DxH800 blood analyzer except coagulation and hemolysis. The following parameters were evaluated: WBC count, neutrophil percentage (NE%), lymphocyte percentage (LY%), monocyte percentage (MO%), and CPD parameters (V, V-SD, C, C-SD, UMALS, UMALS-SD, MALS, MALS-SD, LMALS, LMALS-SD, LALS, LALS-SD, AL2, and AL2-SD).

### 2.3 Statistical methods

The GraphPad Prism 8.0 statistical software (GraphPad Software, San Diego, CA, USA) was used for all data processing. After specimen testing, we obtained the data of all specimens and calculated the average value of testing results for all parameters in different groups. Data were exhibited by the mean ± standard deviation depending on the data characteristics, and the differences were evaluated using the independent sample *t* test in comparisons between two groups. Comparison among three means was performed using the one-way ANOVA (and nonparametric). The parameters with the statistical difference between the ATB and CAP were analyzed by the receiver operating characteristic (ROC) curve and appraised the diagnostic value. The cutoff value was defined based on the max You-den’s index. A *p*-value <0.05 was considered a statistically significant difference.

## 3 Results

### 3.1 Basic information of each parameter

This study retrospectively analyzed 88 patients with ATB (mean age 49.8 ± 14.5 years; male/female: 42/46 years), 78 patients with community-acquired pneumonia (age: 50.9 ± 19.1 years; male/female: 35/43 years), and 89 healthy controls (age: 51.9 ± 19.1 years; male/female: 41/48 years). The values of the following 27 indicators were significantly higher in the ATB group compared with the CAP group (LY%, MO%, NMV-SD, NMC, NMC-SD, N-MALS, N-MALS-SD, N-UMALS, N-LMALS-SD, N-LALS-SD, N-AL2, LMV-SD, LMC, LMC-SD, L-MALS,L-MALS-SD, L-UMALS, L-UMALS-SD, L-LMALS-SD, L-LALS-SD, L-AL2-SD, MMV, MMV-SD, MMC, MMC-SD, M-MALS, M-UMALS; *P* < 0.05). The values of 11 parameters (WBC, NE%, N-LALS, L-AL2, M-MALS-SD, M-UMALS-SD, M-LMALS-SD, M-LALS, M-LALS-SD, M-AL2, and M-AL2-SD; *P* < 0.05) was significantly lower in the ATB group than in the CAP group, while the values of NMV, N-UMALS-SD, N-LMALS, N-AL2-SD, LMV, L-LMALS, L-LALS, and M-LMALS were not statistically significantly different between active TB and community-acquired pneumonia groups (Table A1).

### 3.2 Sensitivity and specificity of CPD parameters to identify active TB and community-acquired pneumonia

**Figure 1:**
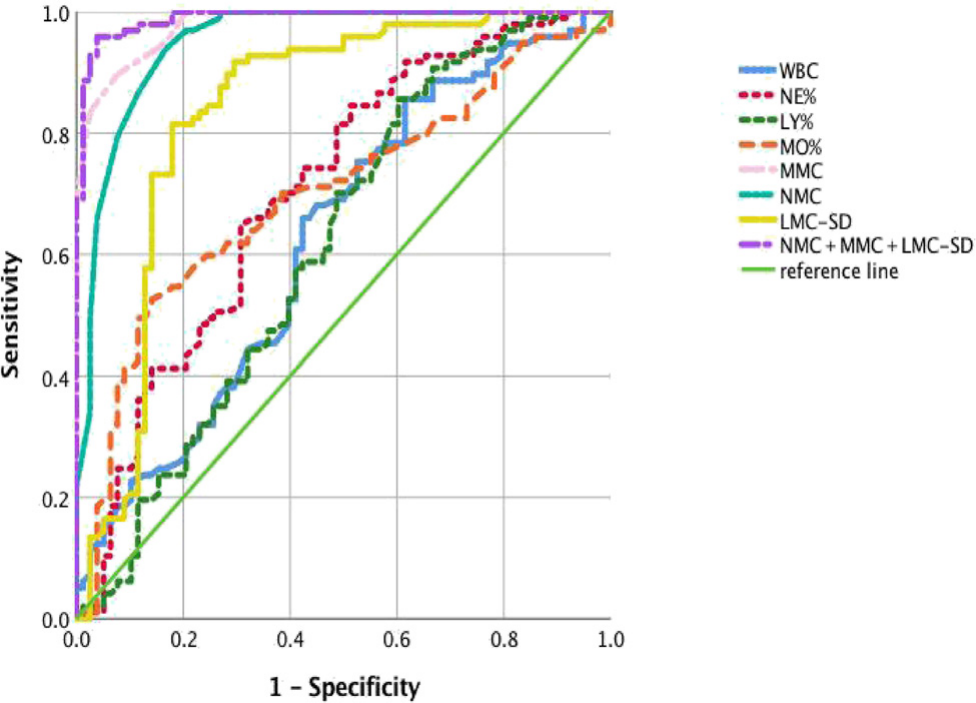
Analysis each parameter between ATB and CAP which under the ROC curve. Diagnostic value was appraised by the receiver operating characteristic (ROC) curve. Cutoff value was defined based on the max You-den’s index, which is the value when the difference between the sensitivity and 1-specificity of all points on the ROC curve is the largest. Compared with the WBC count and three of WBC conventional differential count, the CPD parameter achieved a higher AUC, and the maximum area under the AUC curve obtained by combining MNC, MMV, and MMC was 0.99. WBC, white blood cells; NE%, neutrophil percentage; LY%, lymphocyte percentage; MO%, monocyte percentage; NMC, neutrophil mean conductivity; MMV, monocyte mean volume; MMC, monocyte mean conductivity.

The area under the ROC curve of CPD parameters had significant differences between ATB and CAP groups. WBC, NE%, LY%, and MO% did not show high sensitivity and specificity, and their area under the curve (AUC, 0.63, 0.71, 0.62, and 0.7) was less than 0.75 (Table 1). Conversely, in the analysis of CPD parameters, there are 13 parameters with AUC area above 0.8 (Table A2), of which three parameters (NMC, MMV, and MMC) achieved higher AUC compared with other parameters, which were 0.951, 0.877, and 0.98, respectively (Table 1). Combined measurement of NMC, MMV, and MMC could achieve an AUC as high as 0.99, and its sensitivity and specificity to distinguish ATB from CAP group were 98.5% and 91.1%, respectively (Table 1 and Figure 1).

**Table 1:** Sensitivity and specificity of cut-off values for each parameter in the analysis of active tuberculosis and community-acquired pneumonia groups

Parameters	AUC	Cut-off value	Sensitivity (%)	Specificity (%)
WBC	0.63	>8.450	38.46	85.57
NE%	0.71	>66.70	69.23	64.95
LY%	0.62	<13.08	39.74	85.57
MO%	0.70	<9.550	85.9	52.58
NMC	0.951	<149.5	83.33	93.81
MMV	0.877	<169.5	71.79	89.69
MMC	0.98	<129.5	92.31	89.69
NMC + MMV + MMC	0.99	—	98.5	91.1

WBC: white blood cells, NE%: neutrophil percentage, LY%: lymphocyte percentage, MO%: monocyte percentage, NMC: neutrophil mean conductivity, MMV: monocyte mean volume, MMC: monocyte mean conductivity.

## 4 Discussion

Pneumonia is an infectious disease characterized by the inflammation of the terminal airways, alveoli, and interstitial lungs, affecting people worldwide, including developed and developing countries. In Asian countries, 1 million adults are affected by community-acquired pneumonia alone [7]. Pneumonia can be caused by pathogenic microorganisms, immune damage, physical and chemical factors, allergies, and drugs. The common pathogens of infectious pneumonia include bacteria, viruses, mycoplasma, fungi, rickettsia, chlamydia, and protozoa. Among these, bacterial pneumonia is the most common cause of pneumonia, accounting for 80% of all types of adult infectious pneumonia. Globally, pneumonia is a serious public health concern and a major cause of mortality and morbidity [8]. In recent years, with the aging of the population, the abuse of antibacterial drugs, the increase in immune damage, and the changes in the human living environment have led to great difficulty in the diagnosis and treatment of pneumonia [9,10,11,12]. Rapid and accurate diagnosis, timely treatment and management of the patients, and proper use of antibiotics need urgent attention.

TB has long been a global health problem. According to WHO global tuberculosis report in 2019 that there were 10 million new people suffer from TB, and the number of deaths caused by TB has been higher than that caused by HIV/AIDS in the last 5 years. China is 1 of the 22 countries with the largest TB epidemic burden in the world; the number of patients with TB is second to that of India [13]. However, if diagnosed and treated correctly, most patients with TB can be cured. The diagnosis of TB is mainly based on medical history, clinical manifestations, chest X-ray examination, and examination of sputum TB bacteria; however, the bacteriological examination is time consuming [14,15]. The clinical manifestations of TB, such as cough, fever, and the diversity of chest X-ray results, account for the lack of specificity. Hence, it is easily confused with community-acquired pneumonia, leading to the failure to diagnose and treat in time.

In this study, the VCS technology of the UniCel DxH800 blood analyzer was used to directly examine the peripheral blood of patients with active TB and community-acquired pneumonia. The results confirmed statistically significant differences in NMC, MMV, and MMC between the two groups (*P* < 0.0001; Table 1 and Figure 1). These three parameters were combined to obtain the area under the ROC curve of 0.99, the sensitivity and specificity were 98.5% and 91.1%, respectively. Compared with the WBC count and WBC differential percentage, the combination of NMC, MMV, and MMC had higher sensitivity and specificity with higher diagnostic efficacy for distinguishing ATB from CAP.

Studies have shown that phagocytes, such as monocytes, macrophages, and multinuclear neutrophils, are the first line of defense against the invasion of bacterial pathogens. During bacterial invasion, macrophages are identified by surface exposure, and vesicle or cytoplasmic pattern recognition receivers recognize bacteria and initiate phagocytic cells for phagocytosis. Neutrophils leave the circulation and migrate to inflammatory lesions to deal with the infection. Therefore, after the body is infected by bacteria, there are changes in the size of the white blood cell subpopulation, the number of particles, the nuclear-to-cytoplasmic ratio, and the cell membrane. These differences might vary in different diseases. The results of this study showed that among all CPD parameters, neutrophils, and monocytes, had statistically significant differences in the identification of active TB and community-acquired pneumonia. These changes indicated that the volume of activated cells and volume heterogeneity increase after infection. The nuclear/cytoplasmic ratio was high (the nucleus was more naïve). In the anti-TB immune response, various cytokines are secreted, the number of particles increases in the cytoplasm, and the membrane morphology changes, resulting in a significant change in the electrical conductivity of the cells.

In conclusion, leukocyte community parameters were easily obtained from blood CPD parameters, and the detection cost was low. These parameters could quickly, accurately, and objectively reflect the inherent biological characteristics of cells, thus having potential clinical application value in providing quick and easy reference indicators of ATB and CAP.

## Appendix

**Table A1:** parameters in active tuberculosis and community-acquired pneumonia and healthy control (Mean ± SD)

Parameter	1	2	3	*P*		
	ATB (*N* = 88)	CAP (*N* = 78)	HC (*N* = 89)	1 vs 2	1 vs 3	2 vs 3
WBC	6.614 ± 2.224	8.175 ± 4.097	7.086 ± 2.746	0.0208	Ns	Ns
NE%	64.18 ± 9.191	71.31 ± 13.1	68.74 ± 12.46	<0.0001	0.0075	Ns
LY%	20.7 ± 8.047	18.04 ± 13.6	21.23 ± 10.25	0.0302	Ns	0.0348
MO%	9.899 ± 3.522	7.945 ± 2.992	7.466 ± 2.579	<0.0001	<0.0001	Ns
neutrophil CPD parameter						
NMV	144.2 ± 6.266	145.2 ± 7.995	145.7 ± 7.233	Ns	Ns	Ns
NMV-SD	20.32 ± 3.177	18.81 ± 2.633	17.85 ± 2.107	0.0059	<0.0001	Ns
NMC	153.4 ± 2.645	139.8 ± 7.314	151.5 ± 3.526	<0.0001	0.0003	<0.0001
NMC-SD	9.042 ± 3.948	5.863 ± 1.734	5.536 ± 1.051	<0.0001	<0.0001	Ns
N-MALS	134.3 ± 5.684	128.99.347	136.6 ± 4.945	0.0006`	0.0262	<0.0001
N-MALS-SD	14.95 ± 5.118	12.73 ± 2.612	11.05 ± 1.572	0.0305	<0.0001	<0.0001
N-UMALS	137.8 ± 4.474	124.2 ± 11.22	136.3 ± 5.342	<0.0001	Ns	<0.0001
N-UMALS-SD	14.5 ± 4.385	16.82 ± 6.577	11.85 ± 1.68	Ns	<0.0001	<0.0001
N-LMALS	126.8 ± 9.128	127.3 ± 11.23	133 ± 6.441	Ns	<0.0001	0.0016
N-LMALS-SD	19.16 ± 6.887	16.26 ± 4.386	14.05 ± 2.624	0.0008	<0.0001	0.0004
N-LALS	155.2 ± 20.28	163.6 ± 18	170.9 ± 13.64	0.011	<0.0001	Ns
N-LALS-SD	41.1 ± 6.712	34.27 ± 7.237	33.03 ± 6.652	<0.0001	<0.0001	Ns
N-AL2	142.8 ± 3.893	140.7 ± 4.248	140.7 ± 5.399	0.0023	0.0048	Ns
N-AL2-SD	15.72 ± 4.343	15.73 ± 3.459	11.56 ± 1.843	Ns	<0.0001	<0.0001
lymphocyte CPD parameter						
LMV	89.19 ± 4.457	89.1 ± 4.323	87.49 ± 3.956	Ns	0.0409	Ns
LMV-SD	17.55 ± 3.883	15.38 ± 2.686	15.31 ± 2.534	0.0011	<0.0001	Ns
LMC	119.6 ± 3.204	111.7 ± 6.74	120.4 ± 3.021	<0.0001	Ns	<0.0001
LMC-SD	13.09 ± 2.558	9.642 ± 3.156	9.136 ± 1.923	<0.0001	<0.0001	Ns
L-MALS	61.27 ± 6.314	51.29 ± 13.23	64.53 ± 7.477	<0.0001	0.0029	<0.0001
L-MALS-SD	19.65 ± 2.614	17.42 ± 1.702	17.59 ± 1.808	<0.0001	<0.0001	Ns
L-UMALS	64.13 ± 8.007	44.54 ± 20.61	66 ± 9.197	<0.0001	Ns	<0.0001
L-UMALS-SD	21.91 ± 2.516	19.99 ± 2.875	19.41 ± 1.508	<0.0001	<0.0001	Ns
L-LMALS	54.03 ± 5.828	51.71 ± 7.47	58.43 ± 6.191	Ns	<0.0001	<0.0001
L-LMALS-SD	21.67 ± 2.818	20.19 ± 1.55	20.36 ± 1.664	<0.0001	<0.0001	Ns
L-LALS	39.85 ± 4.814	38.87 ± 2.894	37.65 ± 2.692	Ns	<0.0001	0.0207
L-LALS-SD	14.94 ± 4.987	12.38 ± 1.96	11.69 ± 1.637	<0.0001	<0.0001	Ns
L-AL2	75.76 ± 3.785	79.28 ± 3.407	75.23 ± 2.807	<0.0001	Ns	<0.0001
L-AL2-SD	12.78 ± 3.313	11.91 ± 2.354	10.94 ± 1.366	0.0223	<0.0001	0.0195
monocyte CPD parameter						
MMV	182.4 ± 11.35	166 ± 10.34	166.8 ± 7.935	<0.0001	<0.0001	Ns
MMV-SD	21.96 ± 2.533	20.51 ± 3.783	18.95 ± 3.034	0.0004	<0.0001	0.0108
MMC	132.5 ± 2.389	119.4 ± 6.561	128.9 ± 3.146	<0.0001	<0.0001	<0.0001
MMC-SD	7.266 ± 3.161	6.811 ± 2.953	6.087 ± 1.876	0.0216	<0.0001	Ns
M-MALS	84.27 ± 3.353	74.19 ± 12.72	86.59 ± 5.088	0.0002	0.0002	<0.0001
M-MALS-SD	10.91 ± 1.396	13.31 ± 2.944	11.74 ± 1.295	<0.0001	<0.0001	0.0012
M-UMALS	94.48 ± 5.368	76.29 ± 18.02	94.69 ± 6.135	<0.0001	Ns	<0.0001
M-UMALS-SD	11.87 ± 1.99	16.48 ± 4.544	12.73 ± 1.858	<0.0001	0.0015	<0.0001
M-LMALS	71.01 ± 2.845	67.46 ± 9.699	75.58 ± 5.032	Ns	<0.0001	<0.0001
M-LMALS-SD	14.07 ± 3.399	16.77 ± 3.094	14.73 ± 1.617	<0.0001	0.0001	<0.0001
M-LALS	81.4 ± 11.04	87.42 ± 14.32	90.58 ± 12.19	0.0001	<0.0001	Ns
M-LALS-SD	23.19 ± 4.195	25.94 ± 3.796	26.43 ± 3.898	<0.0001	<0.0001	Ns
M-AL2	131.6 ± 5.154	134.2 ± 4.587	131.3 ± 4.686	0.0003	Ns	0.0001
M-AL2-SD	12.45 ± 2.246	14.71 ± 3.824	12.64 ± 2.588	<0.0001	Ns	<0.0001

ATB: Active Tuberculosis, CAP: Community-acquired pneumonia, HC: Health control, WBC: White blood count, NE%: neutrophil percentage, LY％: lymphocyte percentage, MO%: monocyte percentage, NMV: Neutrophil mean volume,NMV-SD: Neutrophil mean volume standard deviation, NMC: Neutrophil mean conductivity, NMC-SD: Neutrophil mean conductivity standard deviation, N-MALS: Neutrophil medium angle light scatter, N-MALS-SD: Neutrophil medium angle light scatter standard deviation: N-UMALS: Neutrophil upper medium angle light scatter, N-UMALS-SD: Neutrophil upper medium angle light scatter standard deviation, N-LMALS: Neutrophil lower medium angle light scatter, N-LMALS-SD: Neutrophil lower medium angle light scatter standard deviation, N-LALS: Neutrophil lower angle light scatter, N-LALS-SD: Neutrophil lower angle light scatter standard deviation, N-AL2: Neutrophil axial light loss measurement, N-AL2-SD: Neutrophil axial light loss measurement standard deviation, LMV: Lymphocyte mean volume, LMV-SD: Lymphocyte mean volume standard deviation, LMC: Lymphocyte mean conductivity,LMC-SD: Lymphocyte mean conductivity standard deviation, L-UMALS:Lymphocyte upper median angle light scatter, L-UMALS-SD: Lymphocyte upper median angle light scatter standard deviation, L-MALS: Lymphocyte medium angle light scatter, L-MALS-SD: Lymphocyte medium angle light scatter standard deviation, L-LMALS: Lymphocyte lower median angle light scatter, L-LMALS-SD: Lymphocyte lower median angle light scatter standard deviation, L-LALS: Lymphocyte lower angle light scatter, L-LALS-SD: Lymphocyte lower angle light scatter standard deviation, L-AL2: Lymphocyte axial light loss measurement, L-AL2-SD: Lymphocyte axial light loss measurement standard deviation, MMV: Monocyte mean volume, MMV-SD: Monocyte mean volume standard deviation, MMC: Monocyte mean conductivity, MMC-SD: Monocyte mean conductivity standard deviation, M-UMALS: Monocyte upper median angle light scatter, M-UMALS-SD: Monocyte upper median angle light scatter standard deviation, M-MALS: Monocyte medium angle light scatter, M-MALS-SD: Monocyte medium angle light scatter standard deviation, M-LMALS: Monocyte lower median angle light scatter, M-LMALS-SD: Monocyte lower median angle light scatter standard deviation, M-LALS: Monocyte lower angle light scatter, M-LALS-SD: Monocyte lower angle light scatter standard deviation, M-AL2: Monocyte axial light loss measurement, M-AL2-SD: Monocyte axial light loss measurement standard deviation.

**Table A2:** Sensitivity and specificity at the designated cut-off values of parameters for differentiating active tuberculosis from community-acquired pneumonia

Parameter	AUC	Cutoff value	Sensitivity (%)	Specificity (%)
NMC	0.951	<149.5	83.33	93.81
NMC-SD	0.807	<6.475	79.49	69.07
N-UMALS	0.874	<132.5	75.64	88.66
LMC	0.816	<114.4	64.1	96.91
LMC-SD	0.836	<10.88	82.05	81.44
L-MALS-SD	0.809	<18.04	76.92	79.38
L-AL2	0.803	>76.5	83.33	64.95
MMV	0.877	<169.5	71.79	89.69
MMC	0.98	<129.5	92.31	89.69
M-MALS-SD	0.806	>11.82	66.67	84.54
M-UMALS	0.803	<87.5	65.38	97.94
M-UMALS-SD	0.846	>12.29	80.77	74.23
M-LMALS-SD	0.829	>15.06	71.79	86.6
